# A mathematical model of ctDNA shedding predicts tumor detection size

**DOI:** 10.1126/sciadv.abc4308

**Published:** 2020-12-11

**Authors:** Stefano Avanzini, David M. Kurtz, Jacob J. Chabon, Everett J. Moding, Sharon Seiko Hori, Sanjiv Sam Gambhir, Ash A. Alizadeh, Maximilian Diehn, Johannes G. Reiter

**Affiliations:** 1 Canary Center for Cancer Early Detection, Department of Radiology, Stanford University School of Medicine, Palo Alto, CA 94304, USA.; 2 Division of Oncology, Division of Hematology, Department of Medicine, Stanford University School of Medicine, Stanford, CA 94305, USA.; 3 Institute for Stem Cell Biology and Regenerative Medicine, Stanford University School of Medicine, Stanford, CA 94305, USA.; 4 Stanford Cancer Institute, Stanford University School of Medicine, Stanford, CA 94305, USA.; 5 Department of Radiation Oncology, Stanford University School of Medicine, Stanford, CA 94305, USA.; 6 Molecular Imaging Program at Stanford, Department of Radiology, Stanford University School of Medicine, Stanford, CA 94305, USA.; 7 Bio-X Program, Stanford University, Stanford, CA 94305, USA.; 8 Department of Bioengineering and Department of Materials Science and Engineering, Stanford University School of Medicine, Palo Alto, CA 94305, USA.; 9 Department of Biomedical Data Science, Biophysics Program, Stanford University, Stanford, CA 94305, USA.

## Abstract

Early cancer detection aims to find tumors before they progress to an incurable stage. To determine the potential of circulating tumor DNA (ctDNA) for cancer detection, we developed a mathematical model of tumor evolution and ctDNA shedding to predict the size at which tumors become detectable. From 176 patients with stage I to III lung cancer, we inferred that, on average, 0.014% of a tumor cell’s DNA is shed into the bloodstream per cell death. For annual screening, the model predicts median detection sizes of 2.0 to 2.3 cm representing a ~40% decrease from the current median detection size of 3.5 cm. For informed monthly cancer relapse testing, the model predicts a median detection size of 0.83 cm and suggests that treatment failure can be detected 140 days earlier than with imaging-based approaches. This mechanistic framework can help accelerate clinical trials by precomputing the most promising cancer early detection strategies.

## Teaser

A mathematical model of tumor evolution and ctDNA shedding informs new cancer early detection approaches.

## INTRODUCTION

Patients with early-stage cancer are more likely to be cured than patients with advanced-stage cancer (*1*–*4*). For example, the 5-year survival rate of patients with lung cancer who are diagnosed at a localized stage is 57%, while for those diagnosed with distant metastases, it is only 5% (*5*). Unfortunately, only 16% of lung cancers are diagnosed at a localized stage. Recently, multiple studies presented new minimally invasive approaches based on cell-free DNA (cfDNA) to detect cancer from blood samples (*6*–*15*). Most cfDNA in the bloodstream is derived from normal cells, while a small proportion is derived from tumor cells and is known as circulating tumor DNA (ctDNA). Presumably, ctDNA is shed by tumor cells undergoing apoptosis or necrosis (*16*–*18*). Small tumors would therefore be harder to detect because fewer tumor cells undergo cell death and shed ctDNA into the bloodstream.

Previous studies showed that 30 to 100% of symptomatic tumors (mostly larger than 3 cm^3^) can be detected from a 10- to 15-ml blood sample (*8*, *9*, *14*). However, assessing whether blood-based tests can also detect still asymptomatic tumors at sizes smaller than 3 cm^3^ with a sufficiently high specificity to reduce cancer mortality requires elaborate clinical trials with tens of thousands of participants. While such trials are already under way, we lack the mechanistic frameworks necessary to predict the expected size of tumors that would be detected with a given sequencing approach and sampling frequency (*19*). Such frameworks would enable investigators to a priori choose a sequencing and sampling strategy with the highest success probability for a given screening population. For example, how would the performance of a screening test change for a subpopulation with tumors with half as many mutations (e.g., lung cancers of nonsmokers versus smokers)? Motivated by these fundamental questions, we developed a stochastic mathematical model of cancer evolution and biomarker shedding to study the potential and the limitations of blood-based cancer early detection tests across various scenarios. This mechanistic framework will help to predict the performance of ctDNA-based tumor detection approaches and thereby inform and optimize the design of future clinical trials to find cancers earlier.

## RESULTS

### Mathematical model of cancer evolution and ctDNA shedding

**Fig. 1:**
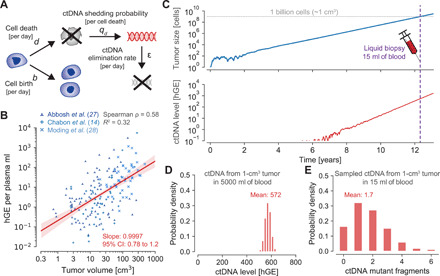
Evolutionary dynamics of ctDNA shed by a growing cancer. (**A**) Mathematical model of cancer evolution and ctDNA shedding. Tumor cells divide with birth rate *b* and die with death rate *d* per day. During cell apoptosis, cells shed ctDNA into the bloodstream with probability *q_d_*. ctDNA is eliminated from the bloodstream with rate ε per day according to the half-life time of ctDNA, *t*_1/2_ = 30 min. (**B**) hGE per plasma ml correlate with tumor volume with a slope of 0.9997 in 176 patients with lung cancer (*R*^2^ = 0.32; *P* = 2.6 × 10^−16^). Red shaded region depicts 95% confidence interval (CI). Linear regression predicts 0.21 hGE per plasma ml for a tumor volume of 1 cm^3^, leading to a shedding probability of *q_d_* = 1.4 × 10^−4^ hGE per cell death (Materials and Methods). (**C**) A tumor starts to grow at time zero with a growth rate of *r* = *b* − *d* = 0.4% (*b* = 0.14, *d* = 0.136), typical for early-stage lung cancers (tumor doubling time of 181 days). Tumor sheds ctDNA into the bloodstream according to the product of the cell death rate *d* and the shedding probability per cell death *q_d_*. (**D**) Distribution of ctDNA hGE in the entire bloodstream at the time [purple dashed line in (C)] when the tumor reaches 1 billion cells [≈1 cm^3^; gray dashed line in (C)], leading to a mean of 0.21 hGE per plasma ml and a tumor fraction of 0.022% at a mean DNA concentration of 6.3 ng per plasma ml. (**E**) Probability distribution of ctDNA mutant fragments present in a liquid biopsy of 15 ml of blood when the tumor reaches 1 billion cells (assuming that the covered somatic heterozygous mutation is present in all cancer cells).

We first consider early-stage lung cancers with a typical tumor volume doubling time of 181 days, leading to a net growth rate of *r* = ln (2)/181 ≈ 0.4% per day (*20*). Lung cancer cells approximately divide with a birth rate of *b* = 0.14 per day (*21*) and die with a death rate of *d* = *b* − *r* = 0.136 per day (Fig. 1A). For now, we assume that each tumor cell releases ctDNA into the bloodstream during apoptosis with a ctDNA shedding probability of *q_d_* per cell death. This assumption implies that the amount of ctDNA linearly correlates with tumor burden, and the slope of the linear regression has to be 1 in logarithmic space.

By reanalyzing ctDNA sequencing data and tumor volumes of 176 patients with stage I to III non–small cell lung cancer of three cohorts (*14*, *22*, *23*), we found that haploid genome equivalents (hGE) per plasma ml indeed correlate with tumor volume with a slope of 0.9997 [95% confidence interval (CI), 0.78 to 1.2; *R*^2^ = 0.32; red line in Fig. 1B; Materials and Methods]. We found similar linear regression slopes and intercepts in the separated three cohorts (fig. S1). For the combined dataset, linear regression predicted 0.21 hGE per plasma ml for 1 cm^3^ of tumor volume (95% CI, 0.15 to 0.28; 95% prediction interval, 0.0033 to 13 hGE per plasma ml for a fixed slope of 1; fig. S2B). On the basis of these analyses, we inferred a mean shedding probability of *q_d_* ≈ 1.4 × 10^−4^ hGE per cell death (95% CI, 1.0 × 10^−4^ to 1.9 × 10^−4^ for a slope of 1; fig. S2). In other words, approximately 0.014% of a cancer cell’s genome is shed into the bloodstream after it undergoes apoptosis. For a ctDNA half-life time of *t*_1/2_ = 30 min (*16*), we calculated a ctDNA elimination rate of ε ≈ 33 per day. We illustrate a typical realization of this evolutionary process in Fig. 1C (movie S1). A tumor grows exponentially with a growth rate of *r* = 0.4% per day and releases ctDNA into the bloodstream with a shedding rate of *d* ∙ *q_d_*. At a primary tumor size of 1 cm^3^ [~1 billion cells (*24*)], we find, on average, 572 ctDNA hGE circulating in the bloodstream (Fig. 1D). A 15-ml blood sample contains approximately 1.7 ctDNA hGE (Fig. 1E). At a mean plasma DNA concentration of 6.3 ng per plasma ml (Materials and Methods), 0.21 ctDNA hGE per plasma ml correspond to a tumor fraction of 0.022% (assuming 6.6 pg per diploid genome). The unit of hGE can also be interpreted as the expected number of ctDNA fragments that exhibit a specific somatic heterozygous mutation. Hence, the number of ctDNA hGE in a sample represents a biological limitation to detect a specific mutation in the tumor. In comparison, given a number of DNA fragments covering a specific genomic region, the number of mutated DNA fragments can be converted to a variant allele frequency (VAF) representing a technological detection limitation due to sequencing errors (*8*, *25*).

Next, we aimed to calculate the expected number of ctDNA haploid genome equivalents, *C*, circulating in the bloodstream for any tumor size *M* and derived a simple closed-form expression. The number of ctDNA hGE circulating when the tumor reaches a size of *M* cells follows a Poisson distribution with a mean of

$$C=M∙d∙q_{d}/(\varepsilon +r)$$ (1)

(for *M* ≫ 1 and *d* ∙ *q_d_* ≪ 1; *r* = *b* − *d*; note S1). For a tumor with 1 billion cells, we calculated a mean of *C* ≈ 572 ctDNA hGE, which perfectly matched the results from the exact computer simulations of the above defined branching process (Fig. 1D). Similarly, for a liquid biopsy of 15 ml of blood (0.3% of 5000 ml), we calculated a mean of 1.7 hGE by multiplying *C* with the fraction of the sampled blood (Fig. 1E).

**Fig. 2:**
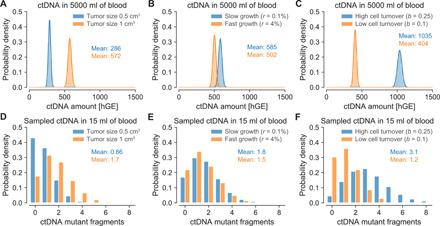
Tumor growth rate and cell turnover strongly affect the amount of ctDNA. (**A** to **C**) ctDNA hGE present in the entire bloodstream when a lung tumor reaches a given size. Bars illustrate distribution of hGE of ctDNA based on 10,000 simulation realizations. Full lines illustrate asymptotic results (Eq. 1; note S1) and perfectly agree with simulation results (bars). (**D** to **F**) ctDNA mutant fragments present in a 15-ml blood sample. (A) Tumors with half the cells (0.5 versus 1 billion cells) lead to half the ctDNA level in the bloodstream (birth rate *b* = 0.14 per cell per day and death rate *d* = 0.136 per cell per day). (B) Assuming that the growth rate proportionally changes the birth and death rates, fast-growing tumors lead to a lower level of ctDNA when they reach a size of 1 billion cells (≈1 cm^3^) because fewer cell deaths decrease the amount of released ctDNA (slow growth: *b* = 0.14 and *d* = 0.139; fast growth: *b* = 0.1595 and *d* = 0.1195). (C) Higher cell turnover rates lead to a higher level of ctDNA at a given tumor size (1 billion cells) compared with lower cell turnover rates because of the increased rate of cells undergoing apoptosis (if the underlying shedding probability per cell death is the same; high cell turnover: *b* = 0.25 and *d* = 0.246; low cell turnover: *b* = 0.1 and *d* = 0.096). Parameter values: ctDNA half-life time *t*_1/2_ = 30 min; ctDNA shedding probability per cell death *q_d_* = 1.4 × 10^−4^ hGE.

To further demonstrate the generality of this framework and the accuracy of our analytical results, we considered tumors with different sizes, growth rates, and cell turnover rates. As expected, a tumor with 0.5 billion cells leads to half the number of circulating biomarkers (*C* ≈ 286 hGE; Fig. 2A). More unexpectedly, a slowly growing lung cancer (*r* = 0.1%) leads to a substantially higher number of 585 hGE than a faster-growing cancer (*r* = 4%) with 502 hGE at the same size of 1 cm^3^, assuming that the faster growth is achieved by proportionally increased birth and decreased death rates (Fig. 2B). If instead the faster growth is achieved by a higher birth rate and an equal death rate, we find a smaller difference (584.2 versus 584.9 hGE). If cancer cells divide with a birth rate of *b* = 0.25 (e.g., colorectal cancer cells), the higher cell turnover rate would lead to a more than twofold (1035 versus 404 hGE) increase in ctDNA compared with a lower cell turnover rate (*b* = 0.1; e.g., breast cancer cells; assuming the same growth rate) because of the increased rate of cells undergoing apoptosis despite the same underlying shedding probability per cell death (Fig. 2C). In general, the tumor growth dynamics, the ctDNA half-life time, and the ctDNA shedding rate strongly influence ctDNA levels (fig. S3). The analytical results were validated by perfectly matching exact simulation results across all considered scenarios (full lines versus bars in the histogram of Fig. 2, A to C; fig. S4 and tables S1 and S2).

Using this mathematical framework of cancer evolution (*26*–*30*), we can predict the expected tumor detection size for an early detection test based on somatic point mutations in ctDNA (*8*, *9*). To compute realistic tumor detection sizes, we considered various sources of biological and technical errors. Given the number of wild-type and mutant fragments, we calculated the probability that a mutation arose from sequencing errors assuming a sequencing error rate of 10^−5^ per base pair (*8*, *25*, *31*). To comprehend the variation of DNA concentration in plasma samples, we reanalyzed previously measured plasma DNA concentrations from Cohen *et al.* (*9*). We used a Gamma distribution with a median of 5.2 ng of DNA per plasma ml (=788 hGE per plasma ml) to model the variability of plasma DNA concentration (fig. S5B; Materials and Methods). This analysis also revealed that plasma DNA concentrations increased in patients with advanced-stage cancer more than expected by the ctDNA amount shed from larger tumors alone (fig. S5E).

We distinguish two types of early cancer detection tests because of their distinct clinical use cases and requirements: (i) cancer screening (somatic mutations of the tumor are not known a priori) and (ii) cancer relapse detection (somatic mutations are known a priori by sequencing a sample of the primary tumor). We start with the fundamentally simpler detection problem (ii) where the mutations are known, and therefore, relatively small custom sequencing panels can be used to detect a relapsing tumor.

### Tumor relapse detection if mutations are known a priori

**Fig. 3:**
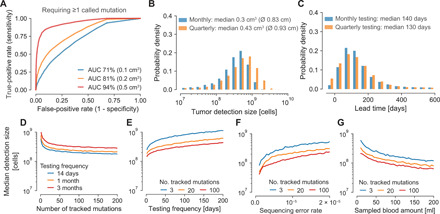
Expected tumor relapse detection size and lead time compared with current clinical relapse detection. (**A**) ROC curves for tumors with 100 million cells (≈0.1 cm^3^; blue line), 200 million cells (≈0.2 cm^3^; orange line), or 500 million cells (≈0.5 cm^3^; red line) when 20 clonal tumor-specific mutations are tracked for relapse detection and one of these 20 mutations needs to be called for a positive test. (**B** to **G**) For better comparability, positive detection test thresholds were set such that if the test is repeated multiple times, a combined FPR of 5% is obtained over all tests per year. (B) Expected tumor detection size distributions for monthly and quarterly repeated relapse detection tests (sequencing panel covers 20 mutations). Ø indicates diameter of spherical tumor. (C) Expected lead time distributions compared with imaging-based approaches applied at the same frequency with a detection limit of 1 cm^3^ for monthly and quarterly repeated relapse detection tests (sequencing panel covers 20 clonal mutations). (D to G) Median tumor detection sizes over the number of clonal mutations covered by the sequencing panel (D), the testing frequency (E), the sequencing error rate (F), and the sampled blood amount (G). Parameter values (if not differently specified): birth rate *b* = 0.14 per cell per day; death rate *d* = 0.13 per cell per day; ctDNA half-life time *t*_1/2_ = 30 min; ctDNA shedding probability per cell death *q_d_* = 1.4 × 10^−4^ hGE; sequencing efficiency of 50%; sequencing error rate per base pair 10^−5^; 15 ml of blood sampled per test; DNA median concentration 5.2 ng per plasma ml. In all scenarios, at least one mutation needs to be called for a positive test. ROC, receiver operating characteristic; AUC, area under the curve.

For cancer relapse detection, we considered an aggressive lung tumor growing with *r* = 1% per day (doubling time of 69 days) and assumed a sequencing panel that covers 20 tumor-specific mutations (*22*, *25*, *32*–*36*). Requiring that at least one of these 20 tumor-specific mutations needs to be called as significantly present in the plasma sample to infer that the tumor relapsed, we find an AUC (area under the curve) for the ROC (receiver operating characteristic) curve of 81% for tumors with 0.2 cm^3^ (diameter of 0.73 cm; Fig. 3A). At a specificity of 99.5%, we observed a sensitivity of 12% for tumors with 0.2 cm^3^, assuming a sequencing efficiency of 50% [i.e., only 50% of the DNA fragments can be assessed (*14*); see fig. S6A for 100% sequencing efficiency]. Repeatedly applying this virtual early detection test to a relapsing lung tumor led to a median detection size of 0.28 cm^3^ for monthly sampling and 0.69 cm^3^ for quarterly sampling (fig. S6D). Important to note is that although the same test with a specificity of 99.5% has been applied for both sampling frequencies, the monthly sampling produces 0.06 false-positive test results over 12 months of relapse testing, while the quarterly sampling only produces 0.02 false positives over 12 months. For an objective comparison, we adjusted the mutation calling thresholds such that 0.05 false positives are expected for both sampling frequencies over 12 months of relapse testing (Materials and Methods). With this adjustment, the median detection size of quarterly testing decreased to 0.43 cm^3^ (Fig. 3B). Monthly relapse testing still led to a 30% smaller median detection size of 0.3 cm^3^ (see fig. S7 for results with 100% sequencing efficiency).

To further assess ctDNA-based relapse detection, we computed the expected lead time to imaging-based relapse detection when applied at the same frequency. We conservatively assumed a radiological detection limit of exactly 1 cm^3^ and a specificity of 100% above that limit. The median lead time of monthly ctDNA testing compared to imaging was 140 days (Fig. 3C). Quarterly ctDNA testing yielded a similar lead time of 130 days because of our assumption that imaging is performed at the same frequency. These predicted lead times for early-stage lung cancer coincide with the reported median of ~160 days by Chaudhuri *et al.* (*37*), the ~120 days by Moding *et al.* (*23*), and the slightly shorter reported median of 70 days (range, 10 to 346 days) by Abbosh *et al.* (*22*), likely due to two required detected mutations, less frequent relapse testing, and a lower average number of clonal mutations covered by the sequencing panel (range, 3 to 26).

Next, we investigated how the sequencing panel size, sampling frequency, blood sample amount, sequencing error, and the number of called mutations for detection affect the tumor detection size. Although we kept the expected number of false positives per year again constant at 0.05, the median tumor detection size strongly decreased up to a sequencing panel size of approximately 25 and then continued to minimally decrease (Fig. 3D). As expected, the median tumor detection size also decreased with an increasing sampling frequency (Fig. 3E). Weekly and more frequent relapse testing led to a large drop of the median detection size. A decreasing sequencing error rate and an increasing amount of sampled blood led to strong decreases of the expected detection sizes (Fig. 3, F and G). Moreover, we found that requiring multiple called mutations for a positive detection test can increase the sensitivity at the same specificity (fig. S8). However, the sensitivity does not increase monotonically with the required number of called mutations and strongly depends on the panel size, plasma DNA concentration, and ctDNA abundance (fig. S9; see Materials and Methods for further details).

### Tumor detection without a priori known mutations

If the mutations in the tumor are not known a priori, cancer detection becomes fundamentally more complex. Two major considerations for ctDNA-based cancer early detection are the expected number of mutations per tumor covered by the sequencing panel and the underlying sequencing error rate per base pair of the assay. The expected number of somatic mutations covered by the sequencing panel can be maximized by focusing on recurrently mutated regions of the genome such that many more mutations per sequenced megabase are observed than expected from the average lung cancer mutation frequency of ~10 mutations per megabase. For example, CAncer Personalized Profiling by deep Sequencing (CAPP-Seq) (spanning ~300,000 base pairs) and CancerSEEK (spanning ~2000 base pairs) cover, on average, 9.1 and 1.1 mutations per early-stage lung cancer, respectively, in the TRACERx cohort (table S3) (*9*, *22*, *25*).

**Fig. 4:**
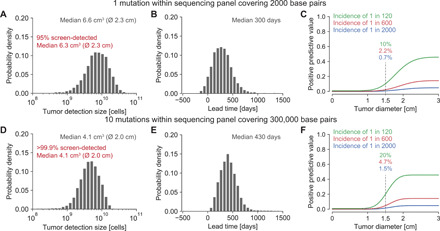
Expected tumor detection size and lead time distributions for screening with different sequencing panels. (**A**) Tumor detection size distribution for annually repeated virtual screening tests with a 2000–base pair sequencing panel covering one somatic mutation per lung cancer. (**B** and **E**) Lead time distributions for annually repeated virtual screening tests compared with current clinical diagnosis times calculated from detection sizes in the SEER database, assuming an early-stage lung cancer growth rate of *r* = 0.4% per day. (**C** and **F**) PPV (positive predictive value) for a cancer early detection test in populations with different lung cancer incidence rates. (**D**) Tumor detection size distribution for annually repeated virtual screening tests with a 300,000–base pair sequencing panel covering 10 somatic mutations per lung cancer of a smoker (table S3). Parameter values typical for early-stage lung cancer: birth rate *b* = 0.14 per cell per day; death rate *d* = 0.136 per cell per day; ctDNA half-life time *t*_1/2_ = 30 min; ctDNA shedding probability *q_d_* = 1.4 × 10^−4^ hGE per cell death; sequencing error rate per base pair 10^−5^; sequencing efficiency 50%; 15 ml of blood sampled per test; plasma normal DNA median concentration 5.2 ng per ml; 99% test specificity.

We again consider an early-stage lung tumor growing with *r* = 0.4% per day. For a sequencing panel covering, on average, one mutation per lung cancer across 2000 base pairs, we computed sensitivities of 4.3, 17, and 54% at a specificity of 99% for tumors at sizes of 1, 2, and 4 cm^3^, respectively. Repeating this virtual early detection test annually for a growing tumor, we obtained a median detection size of 6.6 cm^3^ (diameter of 2.3 cm; Fig. 4A). This detection size would be 70% smaller than the current median detection size of approximately 22.5 cm^3^ (diameter of 3.5 cm; assuming that tumors are approximately spherical) for lung cancers reported in the SEER database from 2005 to 2015 (*38*). Comparing the computed detection size and the SEER median size at diagnosis, we calculated a lead time to current diagnosis times of 300 days for a typical growth rate of early-stage lung cancer (Fig. 4B). For 4.6% of cancers, we observed a negative lead time. In other words, those cancers became symptomatic (i.e., reached typical diagnosis size of 22.5 cm^3^) before they were detected by screening. Faster growing tumors led to larger detection sizes. For example, for more than twice as fast-growing tumors (*r* = 1%, tumor volume doubling time of 69 days), only 52% of cancers would be detected before becoming symptomatic, and their median detection size would be 8.6 cm^3^ (diameter of 2.5 cm; fig. S10). Note that these tumors grow from 0.1 to 3.8 cm^3^ in just 1 year and are therefore very hard to detect with a screening program.

In comparison, for a sequencing panel covering 300,000 base pairs (e.g., CAPP-Seq), we computed sensitivities of 24 and 39% at a specificity of 99% for tumors with a size of 2 cm^3^ in never-smoking subjects and in subjects with a smoking history, respectively. We separately analyzed smokers and nonsmokers because in contrast to the above evaluated panel that focused on driver gene mutations, the expected number of mutations covered by the larger panel for cancers of never-smoking subjects was lower than that for cancers of subjects with a smoking history (5 versus 10 mutations; table S3). For an annually repeated screening test, the expected median detection size was 5.1 cm^3^ (diameter of 2.1 cm) for lung tumors of never-smokers and 4.1 cm^3^ (diameter of 2.0 cm) for tumors of subjects with a smoking history (Fig. 4D). These detection sizes correspond to lead times compared to current tumor sizes at diagnosis of 360 and 430 days for tumors of never-smokers and smokers, respectively (Fig. 4E). Similarly high lead times of up to 450 days were observed in the CCGA (Circulating Cell-free Genome Atlas) study (*15*). In contrast to the informed detection scenario where the sensitivity increased when more than one called mutation was required for detection, in the uninformed detection scenarios, the sensitivity decreased when more than one called mutation was required for detection (fig. S11 versus fig. S9).

For the above calculations, we assumed that the detected mutations in cfDNA were unique to cancers. However, expanded clones in blood or in normal tissue and benign lesions frequently exhibit somatic mutations, which can be observed in cfDNA and hence hamper the specificity of a cancer screening test (*14*, *36*, *39*, *40*). We therefore assumed that white blood cells are sequenced separately to remove somatic mutations arising due to clonal hematopoiesis. Moreover, previous studies showed that, on average, eight benign lung nodules with a diameter of ~0.4 cm exist in subjects undergoing lung cancer screening (*41*). Because most benign lesions are smaller than malignant tumors and because benign cells typically replicate with a lower rate than malignant cells, they are expected to release comparatively less ctDNA into the bloodstream (*21*). Our calculations suggest that these nodules would only slightly increase the expected detection size—even if benign cells exhibit the same ctDNA shedding probability and somatic mutation load as malignant cells (fig. S12).

While these predicted tumor detection sizes are encouraging, a major challenge for every cancer detection test is the low incidence rate of cancers. Relatively common cancers such as lung cancer have yearly incidence rates of approximately 1 in 2000 (*5*). Because more than 98% of lung cancers occur in people above 50 (~30% of the U.S. population), the incidence rate increases to 1 in 600 in this age group. Hence, approximately six false positives would be expected for one true positive at a test specificity of 99%. We calculated a PPV (positive predictive value) of 2.2% and an NPV (negative predictive value) of 99.9% for a lung tumor with a diameter of 1.5 cm using a sequencing panel of 2000 base pairs (Fig. 4C). For heavy smokers in their late 60s, the incidence rate increases to 1 in 120, and the same test would have a PPV of 10% and an NPV of 99.3% for 1.5-cm lung tumors. In comparison, a PPV of 3.8% was reported for low-dose computed tomography (CT) lung cancer screening (*42*).

### ctDNA shedding during apoptosis, necrosis, and proliferation

So far, we assumed that ctDNA shedding occurs exclusively during apoptosis. We can generalize our framework such that the effective shedding rate λ is given by the sum of ctDNA fragments shed during apoptosis, necrosis, and proliferation: λ = *d* ∙ *q_d_* + *q_n_* + *b* ∙ *q_b_*, where *q_b_* denotes the shedding probability per cell division and *q_n_* denotes the shedding rate from necrosis per unit of time (*10*, *16*). We show that independent of the three shedding processes, the amount of ctDNA when the tumor reaches a size of *M* cells remains approximately Poisson distributed with a mean of *C* = *M* ∙ λ/(ε + *r*) (assuming *M* ≫ 1 and λ ≪ 1; note S1), where the effective shedding rate represents the sum of the operating ctDNA shedding processes (fig. S13 and table S1).

## DISCUSSION

Our mathematical framework provides a theoretical estimate for the performance of mutation-based ctDNA detection across clinical scenarios. For example, ctDNA becomes increasingly important for identifying actionable mutations when tumor tissue is not available. On the basis of our stochastic model, we estimate that the probability of a false negative for a particular actionable mutation clonally present in tumors with diameters of 1, 1.5, and 2 cm is 82, 44, and 9.3%, respectively (requiring a specificity of 99%). A critical parameter for these estimates is the ctDNA shedding rate per cancer cell, which can vary by multiple magnitudes among patients (Fig. 1B). The stochastic model indicates that decreasing the sequencing error rate, increasing the amount of sampled plasma, increasing the sequencing panel size, and increasing the sampling rate can drastically decrease the expected tumor relapse detection size at the same normalized annual false-positive rate (FPR) (Fig. 3). The virtual screening computations indicate that lung tumors would be detected when they reach a diameter of 2.3 cm in an annual screening program with a sequencing panel of 2000 base pairs (Fig. 4A). According to the lung cancer staging system for tumor sizes, 12% of the screen-detected cases would be classified as T1a (≤1 cm), 21% as T1b (>1 but ≤2 cm), and 55% as T1c (>2 but ≤3 cm). Only 12% of the screen-detected tumors would have reached sizes for stage T2a (>3 but ≤4 cm) or beyond. Although these calculations suggest that most tumors can only be detected when they reach sizes of billions of cells, detecting some tumors before they become symptomatic and shifting some diagnoses to an earlier stage can have an enormous impact on cancer mortality (*4*, *43*).

This study has several limitations. First, our understanding of ctDNA shedding and its variance across lung tumors and other tumor types remains limited. More studies such as those by Abbosh *et al.*, Chabon *et al.*, and Moding *et al.* correlating ctDNA levels with tumor volume and other clinicopathological features in patients with lung cancer are required to inform shedding rate inferences in other tumor types (*14*, *22*, *23*). The high concordance in both the slope and the intercept of the linear regression analysis across the three aforementioned lung cancer cohorts is reassuring (Fig. 1B and fig. S1). Other biological factors such as tumor histology and stage additionally influence ctDNA levels (*14*) and could be included in future models when more data become available. Second, the ctDNA shedding dynamics of precursor lesions and how their presence interferes with cancer early detection are largely unknown (fig. S12). Third, our analysis was limited to point mutations present in ctDNA. Including additional cancer-associated characteristics of ctDNA or other biomarkers can help to further decrease the expected detection size (*9*–*15*, *17*). For example, the detection of differentially methylated regions or of copy number variants can be modeled with our framework (*11*, *44*). Last, we verified our mathematical results through exact computer simulations; however, the predicted tumor detection sizes will need to be validated in large clinical studies. According to the model, every tumor eventually becomes detectable—but some will become symptomatic first (e.g., those cancers with negative lead times in Fig. 4). We note that tumor-specific ctDNA mutant fragments were detected in all TRACERx subjects, but no mutations were called because of the high specificity requirements in 40% (38 of 96) of subjects (*22*).

A major challenge of cancer early detection is the stochastic nature of cancer initiation and progression. While new technologies can detect smaller and smaller tumors, the optimal treatment of asymptomatic tumors is often unclear and needs to be balanced with the risk for overtreatment and undertreatment (*3*, *4*). For example, in more than 20% of smokers, suspicious lesions can be found by low-dose computed tomographic screening; nevertheless, lung cancer was detected only in ~0.6% of screened smokers within a 10-year follow-up (*45*, *46*). Similarly, around 33% of humans harbor precursor lesions in their pancreas, but the lifetime risk of developing pancreatic cancer is 1.6% (*38*, *47*). Hence, most precursor lesions do not progress to cancer within the lifetime of humans (*39*, *48*). Because cancer incidence strongly depends on factors such as age, genetic predisposition, lifestyle, or exposure to mutagens (e.g., sun, smoke), screening programs often focus on high-risk individuals to decrease the chances of overtreatment. Our results show that cancer screening and surveillance strategies can be further optimized and personalized by comprehensive mathematical models of cancer evolution and biomarker shedding (*19*, *28*, *48*–*51*).

## MATERIALS AND METHODS

### Parameter selection and inference

We used previously measured values of cell division rates and tumor volume doubling times to obtain the tumor growth rates and death rates. The estimated average time between cell divisions of lung cancer cells is 7 days (*21*), resulting in a cell birth rate of *b* = 0.14 per day. Given a volume doubling time of ~180 days of stage I lung cancers (*20*), we find a tumor growth rate *r* = ln (2)/180 ≈ 0.4% per day and a death rate of *d* = *b* − *r* = 0.136 per day. For the analysis of tumors with a high cell turnover rate (Fig. 2C), we assumed an average time between cell divisions of colorectal cancer cells of 4 days (*21*), resulting in a cell birth rate of *b* = 0.25 per day. For tumors with a low cell turnover rate, we assumed an average time between cell divisions of breast cancer cells of 10 days (*21*), resulting in a cell birth rate of *b* = 0.1 per day. For the analysis with coexisting benign lesions (fig. S12), we explored the effects of nodules at fixed sizes due to their doubling times of ≥500 days and assumed that cells in benign lung nodules replicate with rates of *b*_bn_ = *d*_bn_ = 0.07 per day (*20*, *21*).

To estimate the ctDNA shedding rate per cancer cell, we reanalyzed data from 176 patients with stage I to III non–small cell lung cancer studied in Chabon *et al.* (*14*), Abbosh *et al.* (*22*), and Moding *et al.* (*23*). In Chabon *et al.*, metabolic tumor volumes were computed from whole-body ^18^F-FDG PET (positron emission tomography)–CT scans in 81 subjects. For 46 of these subjects, tumor-specific ctDNA mutations were reported. Reanalyzed gross tumor volume from Abbosh *et al.* and Moding *et al.* was estimated from preoperative CT scans. hGE per plasma ml was calculated from the mean VAF times the plasma cfDNA concentration in a subject divided by 0.0033 ng (weight of haploid human genome). Estimates can be found in Supplementary Table 3 of Chabon *et al.* and Supplementary Table 2 of Moding *et al.* For the data in Abbosh *et al.*, the mean VAF of somatic mutations was calculated across all mutations (including those that were not called in the liquid biopsy) that were identified as clonal in the primary tumor [in subject CRUK0053, all mutations were considered because no clonality information was available; see Supplementary Table 5 of (*22*)].

To infer the shedding rate λ, we used the predicted 0.21 hGE per plasma ml for a tumor with a volume of 1 cm^3^ (assuming 1 billion cells per cm^3^; Fig. 1B) and set up the following equilibrium equation of λ ∙ 10^9^ cells = 577.5 ∙ ε, assuming 577.5 hGE (=0.21 ∙ 5000 ∙ 55%) in 5000 ml of blood with a plasma concentration of 55%. We used previous estimates of the ctDNA half-life time of *t*_1/2_ = 30 min and explored the effects of half-life times from 20 to 120 min (fig. S3) (*16*). We calculated the ctDNA elimination rate as ε=ln2t1/2∙60∙24≈33.3 fragments per day. We found a shedding rate of λ = 1.9 ∙ 10^−5^ hGE per cell per day, leading to a shedding probability of qd=λd=1.4×10−4 hGE per cell death, assuming that ctDNA is exclusively shed during cell apoptosis. To estimate the shedding rate of cells in other tissue types, we can rescale the shedding probability by the corresponding cell death rate. For example, we estimate the shedding rate of cells in benign lung nodules as λ_bn_ = *d*_bn_ ∙ *q_d_* ≈ 9.8 ∙ 10^−6^ hGE per cell per day.

We investigated how a distribution of values for the shedding probability *q_d_* would influence our results (fig. S2). Normality tests performed on the residuals of the linear regression between ctDNA levels and tumor volume (Fig. 1B) were consistent with a normal distribution and supported the assumption of a slope 1 for the linear correlation in log-log space. By fixing the slope to 1, we inferred the 95% CI for the amount of ctDNA shed by a tumor of 1 cm^3^ per plasma ml (fig. S2B). Because the CI is symmetric in logarithmic scale with respect to the predicted value of 0.21 hGE per plasma ml, we modeled the variability of this parameter by fitting a normal distribution in log space to match the estimated 95% CI (fig. S2C). Through our equilibrium equation and estimated death rate of cancer cells, we converted this normal probability distribution into a lognormal density for the shedding probability *q_d_* in linear space [mean, 1.4 × 10^−4^ hGE per cell death; SD (standard deviation), 2.2 × 10^−5^]. The average amount of ctDNA mutant fragments found in a 15-ml blood sample when the shedding probability follows a lognormal distribution is almost the same than when the shedding probability is fixed to the mean of the lognormal distribution (1.74 hGE versus 1.72 hGE; fig. S2E). To facilitate the analysis and interpretation of ctDNA shedding for cancer early detection, we assumed a fixed value of *q_d_*.

### In silico sampling and sequencing of plasma DNA

To compare different screening strategies, we used virtual sampling and sequencing of DNA. For each liquid biopsy, ctDNA fragments in 15 ml of blood were sampled from 5000 ml of blood according to a binomial distribution. For the virtual detection tests in Figs. 3 and 4 and figs. S6 to S12, plasma cfDNA concentrations were sampled from a Gamma distribution (mean and median of 6.3 and 5.2 ng per plasma ml, respectively) as illustrated in fig. S5B. We assumed that 50% of cfDNA fragments are assessed by sequencing (except in figs. S6, A to C, and S7). To calculate the cfDNA genome equivalents, we assumed a weight of 6.6 pg per diploid genome (*17*). The sequencing error rate per base pair was set to *e*_seq_ = 10^−5^ (*8*, *25*, *31*). For each mutation covered by the sequencing panel, we account for sequencing errors by calculating a *P* value as $1-\Sigma_{i=0}^{k-1}(\begin{array}{c} n\\ i \end{array})\cdot {e_{\text{seq}}}^{i}\cdot {\left(1-e_{\text{seq}}\right)}^{n-i}$, where *k* denotes the number of observed fragments supporting the mutation, and *n* denotes the total number of fragments covering the mutation’s genomic position.

To optimize the performance of ctDNA-based cancer detection, we calculated the sensitivity and specificity when more than one called mutation is required for detection. These calculations show a nonmonotonically changing sensitivity for different numbers of called mutation thresholds to classify a test as positive (fig. S9C). This counterintuitive behavior is ascribable to the mutation calling *P* value threshold, which we adapt to keep the FPR approximately constant to objectively compare different testing approaches (fig. S9, A and B). The estimated sensitivity for a fixed plasma normal DNA concentration and a fixed ctDNA level changes according to a reverse sawtooth wave for an FPR of ≤1%, a sequencing error rate of 10^−5^, and a sequencing panel size of 20 where all mutations are clonally present in a relapsing tumor (fig. S9D). The sensitivity jumps occur at different numbers of required mutations for detection for the different plasma DNA concentrations. For example, for a plasma DNA concentration of 12.4 ng/ml (yellow curve in fig. S9D), we observe two jumps: (i) when instead of 3, only 2 mutant fragments become sufficient to reach a *P* value below the threshold for the desired FPR, and (ii) when instead of 2, only 1 mutant fragment becomes sufficient to reach a *P* value below the threshold for the given FPR. When the whole distribution of plasma DNA concentration is taken into account (fig. S5B), the sensitivity varies more smoothly but still increases locally when the number of mutant fragments required jumps downward (fig. S9E). These local maxima are also affected by the ctDNA amount (fig. S9F). Assuming 50 ctDNA hGE in the bloodstream, the sensitivity reaches a single maximum value (at 4 mutations required for detection) before decreasing to almost zero (blue curve). At a value of 100 ctDNA hGE, the sensitivity shows two local maxima, which shift as the amount of ctDNA increases. For instance, peaks are at 2 and 6 called mutations required for detection with 100 ctDNA hGE and at 3 and 9 called mutations with 300 ctDNA hGE. With 600 ctDNA hGE or more, the sensitivity saturates and stays constant at almost 100% before starting to decrease monotonically. These results can then be used to study sensitivity as a function of tumor sizes (and hence of varying ctDNA levels). For an FPR of 1% and tumor diameters between roughly 0.73 cm (2 × 10^8^ cells) and 1.0 cm (6 × 10^8^ cells), 8 called mutations required for detection led to a higher sensitivity than 2, 4, and 1 mutations (in decreasing order; fig. S9G).

## Supplementary Material

http://advances.sciencemag.org/cgi/content/full/6/50/eabc4308/DC1

Movie S1

Adobe PDF - abc4308_SM.pdf

A mathematical model of ctDNA shedding predicts tumor detection size

## SUPPLEMENTARY MATERIALS

Supplementary material for this article is available at http://advances.sciencemag.org/cgi/content/full/6/50/eabc4308/DC1

## References

[1] Etzioni R., Urban N., Ramsey S., McIntosh M., Schwartz S., Reid B., Radich J., Anderson G., Hartwell L., Early detection: The case for early detection. Nat. Rev. Cancer 3, 243–252 (2003). 12671663 10.1038/nrc1041

[2] Song M., Vogelstein B., Giovannucci E. L., Willett W. C., Tomasetti C., Cancer prevention: Molecular and epidemiologic consensus. Science 361, 1317–1318 (2018). 30262488 10.1126/science.aau3830 PMC6260589

[3] Srivastava S., Koay E. J., Borowsky A. D., De Marzo A. M., Ghosh S., Wagner P. D., Kramer B. S., Cancer overdiagnosis: A biological challenge and clinical dilemma. Nat. Rev. Cancer 19, 349–358 (2019). 31024081 10.1038/s41568-019-0142-8 PMC8819710

[4] Mattox A. K., Bettegowda C., Zhou S., Papadopoulos N., Kinzler K. W., Vogelstein B., Applications of liquid biopsies for cancer. Sci. Transl. Med. 11, eaay1984 (2019). 31462507 10.1126/scitranslmed.aay1984

[5] Siegel R. L., Miller K. D., Jemal A., Cancer statistics, 2020. CA Cancer J. Clin. 70, 7–30 (2020). 31912902 10.3322/caac.21590

[6] Bettegowda C., Sausen M., Leary R. J., Kinde I., Wang Y., Agrawal N., Bartlett B. R., Wang H., Luber B., Alani R. M., Antonarakis E. S., Azad N. S., Bardelli A., Brem H., Cameron J. L., Lee C. C., Fecher L. A., Gallia G. L., Gibbs P., Le D., Giuntoli R. L., Goggins M., Hogarty M. D., Holdhoff M., Hong S.-M., Jiao Y., Juhl H. H., Kim J. J., Siravegna G., Laheru D. A., Lauricella C., Lim M., Lipson E. J., Marie S. K. N., Netto G. J., Oliner K. S., Olivi A., Olsson L., Riggins G. J., Sartore-Bianchi A., Schmidt K., Shih l.-M., Oba-Shinjo S. M., Siena S., Theodorescu D., Tie J., Harkins T. T., Veronese S., Wang T.-L., Weingart J. D., Wolfgang C. L., Wood L. D., Xing D., Hruban R. H., Wu J., Allen P. J., Max Schmidt C., Choti M. A., Velculescu V. E., Kinzler K. W., Vogelstein B., Papadopoulos N., Diaz L. A. Jr., Detection of circulating tumor DNA in early- and late-stage human malignancies. Sci. Transl. Med. 6, 224ra24 (2014). 10.1126/scitranslmed.3007094 PMC4017867 24553385

[7] Newman A. M., Bratman S. V.; J. To, Wynne J. F., Eclov N. C. W., Modlin L. A., Liu C. L., Neal J. W., Wakelee H. A., Merritt R. E., Shrager J. B., Loo B. W. Jr., Alizadeh A. A., Diehn M., An ultrasensitive method for quantitating circulating tumor DNA with broad patient coverage. Nat. Med. 20, 548–554 (2014). 24705333 10.1038/nm.3519 PMC4016134

[8] Phallen J., Sausen M., Adleff V., Leal A., Hruban C., White J., Anagnostou V., Fiksel J., Cristiano S., Papp E., Speir S., Reinert T., Orntoft M.-B. W., Woodward B. D., Murphy D., Parpart-Li S., Riley D., Nesselbush M., Sengamalay N., Georgiadis A., Li Q. K., Madsen M. R., Mortensen F. V., Huiskens J., Punt C., van Grieken N., Fijneman R., Meijer G., Husain H., Scharpf R. B., Diaz L. A. Jr., Jones S., Angiuoli S., Ørntoft T., Nielsen H. J., Andersen C. L., Velculescu V. E., Direct detection of early-stage cancers using circulating tumor DNA. Sci. Transl. Med. 9, eaan2415 (2017). 28814544 10.1126/scitranslmed.aan2415 PMC6714979

[9] Cohen J. D., Li L., Wang Y., Thoburn C., Afsari B., Danilova L., Douville C., Javed A. A., Wong F., Mattox A., Hruban R. H., Wolfgang C. L., Goggins M. G., Molin M. D., Wang T.-L., Roden R., Klein A. P., Ptak J., Dobbyn L., Schaefer J., Silliman N., Popoli M., Vogelstein J. T., Browne J. D., Schoen R. E., Brand R. E., Tie J., Gibbs P., Wong H.-L., Mansfield A. S., Jen J., Hanash S. M., Falconi M., Allen P. J., Zhou S., Bettegowda C., Diaz L. A. Jr., Tomasetti C., Kinzler K. W., Vogelstein B., Lennon A. M., Papadopoulos N., Detection and localization of surgically resectable cancers with a multi-analyte blood test. Science 357, 926–930 (2018). 10.1126/science.aar3247 PMC6080308 29348365

[10] Mouliere F., Chandrananda D., Piskorz A. M., Moore E. K., Morris J., Ahlborn L. B., Mair R., Goranova T., Marass F., Heider K., Wan J. C. M., Supernat A., Hudecova I., Gounaris I., Ros S., Jimenez-Linan M., Garcia-Corbacho J., Patel K., Østrup O., Murphy S., Eldridge M. D., Gale D., Stewart G. D., Burge J., Cooper W. N., van der Heijden M. S., Massie C. E., Watts C., Corrie P., Pacey S., Brindle K. M., Baird R. D., Mau-Sørensen M., Parkinson C. A., Smith C. G., Brenton J. D., Rosenfeld N., Enhanced detection of circulating tumor DNA by fragment size analysis. Sci. Transl. Med. 10, eaat4921 (2018). 30404863 10.1126/scitranslmed.aat4921 PMC6483061

[11] Shen S. Y., Singhania R., Fehringer G., Chakravarthy A., Roehrl M. H. A., Chadwick D., Zuzarte P. C., Borgida A., Wang T. T., Li T., Kis O., Zhao Z., Spreafico A., Medina T. d. S., Wang Y., Roulois D., Ettayebi I., Chen Z., Chow S., Murphy T., Arruda A., O’Kane G. M., Liu J., Mansour M., McPherson J. D., O’Brien C., Leighl N., Bedard P. L., Fleshner N., Liu G., Minden M. D., Gallinger S., Goldenberg A., Pugh T. J., Hoffman M. M., Bratman S. V., Hung R. J., de Carvalho D. D., Sensitive tumour detection and classification using plasma cell-free DNA methylomes. Nature 563, 579–583 (2018). 30429608 10.1038/s41586-018-0703-0

[12] Cristiano S., Leal A., Phallen J., Fiksel J., Adleff V., Bruhm D. C., Jensen S. Ø., Medina J. E., Hruban C., White J. R., Palsgrove D. N., Niknafs N., Anagnostou V., Forde P., Naidoo J., Marrone K., Brahmer J., Woodward B. D., Husain H., van Rooijen K. L., Ørntoft M. B. W., Madsen A. H., van de Velde C. J. H., Verheij M., Cats A., Punt C. J. A., Vink G. R., van Grieken N. C. T., Koopman M., Fijneman R. J. A., Johansen J. S., Nielsen H. J., Meijer G. A., Andersen C. L., Scharpf R. B., Velculescu V. E., Genome-wide cell-free DNA fragmentation in patients with cancer. Nature 570, 385–389 (2019). 31142840 10.1038/s41586-019-1272-6 PMC6774252

[13] Ulz P., Thallinger G. G., Auer M., Graf R., Kashofer K., Jahn S. W., Abete L., Pristauz G., Petru E., Geigl J. B., Heitzer E., Speicher M. R., Inferring expressed genes by whole-genome sequencing of plasma DNA. Nat. Genet. 48, 1273–1278 (2016). 27571261 10.1038/ng.3648

[14] Chabon J. J., Hamilton E. G., Kurtz D. M., Esfahani M. S., Moding E. J., Stehr H., Schroers-Martin J., Nabet B. Y., Chen B., Chaudhuri A. A., Liu C. L., Hui A. B., Jin M. C., Azad T. D., Almanza D., Jeon Y. J., Nesselbush M. C., Co Ting Keh L., Bonilla R. F., Yoo C. H., Ko R. B., Chen E. L., Merriott D. J., Massion P. P., Mansfield A. S., Jen J., Ren H. Z., Lin S. H., Costantino C. L., Burr R., Tibshirani R., Gambhir S. S., Berry G. J., Jensen K. C., West R. B., Neal J. W., Wakelee H. A., Loo B. W. Jr., Kunder C. A., Leung A. N., Lui N. S., Berry M. F., Shrager J. B., Nair V. S., Haber D. A., Sequist L. V., Alizadeh A. A., Diehn M., Integrating genomic features for non-invasive early lung cancer detection. Nature 580, 245–251 (2020). 32269342 10.1038/s41586-020-2140-0 PMC8230734

[15] Liu M. C., Oxnard G. R., Klein E. A., Swanton C., Seiden M. V.; CCGA Consortium; STRIVE investigators; GRAIL Inc.; Advisors, Sensitive and specific multi-cancer detection and localization using methylation signatures in cell-free DNA. Ann. Oncol. 31, 745–759 (2020). 10.1016/j.annonc.2020.02.011 PMC8274402 33506766

[16] Wan J. C. M., Massie C., Garcia-Corbacho J., Mouliere F., Brenton J. D., Caldas C., Pacey S., Baird R., Rosenfeld N., Liquid biopsies come of age: Towards implementation of circulating tumour DNA. Nat. Rev. Cancer 17, 223–238 (2017). 28233803 10.1038/nrc.2017.7

[17] Heitzer E., Haque I. S., Roberts C. E. S., Speicher M. R., Current and future perspectives of liquid biopsies in genomics-driven oncology. Nat. Rev. Genet. 20, 71–88 (2019). 30410101 10.1038/s41576-018-0071-5

[18] Schwarzenbach H., Hoon D. S. B., Pantel K., Cell-free nucleic acids as biomarkers in cancer patients. Nat. Rev. Cancer 11, 426–437 (2011). 21562580 10.1038/nrc3066

[19] Pashayan N., Pharoah P. D. P., The challenge of early detection in cancer. Science 368, 589–590 (2020). 32381710 10.1126/science.aaz2078

[20] Winer-Muram H. T., Jennings S. G., Tarver R. D., Aisen A. M., Tann M., Conces D. J., Meyer C. A., Volumetric growth rate of stage I lung cancer prior to treatment: Serial CT scanning. Radiology 223, 798–805 (2002). 12034952 10.1148/radiol.2233011026

[21] Rew D. A., Wilson G. D., Cell production rates in human tissues and tumours and their significance. Part II: clinical data. Eur. J. Surg. Oncol. 26, 405–417 (2000). 10873364 10.1053/ejso.1999.0907

[22] Abbosh C., Birkbak N. J., Wilson G. A., Jamal-Hanjani M., Constantin T., Salari R., Le Quesne J., Moore D. A., Veeriah S., Rosenthal R., Marafioti T., Kirkizlar E., Watkins T. B. K., Granahan N. M., Ward S., Martinson L., Riley J., Fraioli F., Bakir M. A., Grönroos E., Zambrana F., Endozo R., Bi W. L., Fennessy F. M., Sponer N., Johnson D., Laycock J., Shafi S., Czyzewska-Khan J., Rowan A., Chambers T., Matthews N., Turajlic S., Hiley C., Lee S. M., Forster M. D., Ahmad T., Falzon M., Borg E., Lawrence D., Hayward M., Kolvekar S., Panagiotopoulos N., Janes S. M., Thakrar R., Ahmed A., Blackhall F., Summers Y., Hafez D., Naik A., Ganguly A., Kareht S., Shah R., Joseph L., Quinn A. M., Crosbie P. A., Naidu B., Middleton G., Langman G., Trotter S., Nicolson M., Remmen H., Kerr K., Chetty M., Gomersall L., Fennell D. A., Nakas A., Rathinam S., Anand G., Khan S., Russell P., Ezhil V., Ismail B., Irvin-Sellers M., Prakash V., Lester J. F., Kornaszewska M., Attanoos R., Adams H., Davies H., Oukrif D., Akarca A. U., Hartley J. A., Lowe H. L., Lock S., Iles N., Bell H., Ngai Y., Elgar G., Szallasi Z., Schwarz R. F., Herrero J., Stewart A., Quezada S. A., Peggs K. S., Van Loo P., Dive C., Jimmy Lin C., Rabinowitz M., Aerts H. J. W. L., Hackshaw A., Shaw J. A., Zimmermann B. G.; The TRACERx consortium; The PEACE consortium, Swanton C., Phylogenetic ctDNA analysis depicts early-stage lung cancer evolution. Nature 545, 446–451 (2017). 28445469 10.1038/nature22364 PMC5812436

[23] Moding E. J., Liu Y., Nabet B. Y., Chabon J. J., Chaudhuri A. A., Hui A. B., Bonilla R. F., Ko R. B., Yoo C. H., Gojenola L., Jones C. D., He J., Qiao Y., Xu T., Heymach J. V., Tsao A., Liao Z., Gomez D. R., Das M., Padda S. K., Ramchandran K. J., Neal J. W., Wakelee H. A., Loo B. W. Jr., Lin S. H., Alizadeh A. A., Diehn M., Circulating tumor DNA dynamics predict benefit from consolidation immunotherapy in locally advanced non-small-cell lung cancer. Nat. Cancer. 1, 176–183 (2020). 10.1038/s43018-019-0011-0 PMC8425388 34505064

[24] Hori S. S., Gambhir S. S., Mathematical model identifies blood biomarker–based early cancer detection strategies and limitations. Sci. Transl. Med. 3, 109ra116 (2011). 10.1126/scitranslmed.3003110 PMC3423335 22089452

[25] Newman A. M., Lovejoy A. F., Klass D. M., Kurtz D. M., Chabon J. J., Scherer F., Stehr H., Liu C. L., Bratman S. V., Say C., Zhou L., Carter J. N., West R. B., Sledge G. W. Jr., Shrager J. B., Loo B. W. Jr., Neal J. W., Wakelee H. A., Diehn M., Alizadeh A. A., Integrated digital error suppression for improved detection of circulating tumor DNA. Nat. Biotechnol. 34, 547–555 (2016). 27018799 10.1038/nbt.3520 PMC4907374

[26] R. Durrett, *Branching Process Models of Cancer* (Springer, 2015).

[27] D. Wodarz, N. L. Komarova, *Dynamics of Cancer: Mathematical Foundations of Oncology* (World Scientific Publishing, Singapore, 2014).

[28] Reiter J. G., Makohon-Moore A. P., Gerold J. M., Heyde A., Attiyeh M. A., Kohutek Z. A., Tokheim C. J., Brown A., DeBlasio R. M., Niyazov J., Zucker A., Karchin R., Kinzler K. W., Iacobuzio-Donahue C. A., Vogelstein B., Nowak M. A., Minimal functional driver gene heterogeneity among untreated metastases. Science 361, 1033–1037 (2018). 30190408 10.1126/science.aat7171 PMC6329287

[29] Beerenwinkel N., Schwarz R. F., Gerstung M., Markowetz F., Cancer evolution: Mathematical models and computational inference. Syst. Biol. 64, e1–e25 (2015). 25293804 10.1093/sysbio/syu081 PMC4265145

[30] Altrock P. M., Liu L. L., Michor F., The mathematics of cancer: Integrating quantitative models. Nat. Rev. Cancer 15, 730–745 (2015). 26597528 10.1038/nrc4029

[31] Wan J. C. M., Heider K., Gale D., Murphy S., Fisher E., Mouliere F., Ruiz-Valdepenas A., Santonja A., Morris J., Chandrananda D., Marshall A., Gill A. B., Chan P. Y., Barker E., Young G., Cooper W. N., Hudecova I., Marass F., Mair R., Brindle K. M., Stewart G. D., Abraham J. E., Caldas C., Rassl D. M., Rintoul R. C., Alifrangis C., Middleton M. R., Gallagher F. A., Parkinson C., Durrani A., Dermott U. M., Smith C. G., Massie C., Corrie P. G., Rosenfeld N., ctDNA monitoring using patient-specific sequencing and integration of variant reads. Sci. Transl. Med. 12, eaaz8084 (2020). 32554709 10.1126/scitranslmed.aaz8084

[32] McDonald B. R., Contente-Cuomo T., Sammut S.-J., Odenheimer-Bergman A., Ernst B., Perdigones N., Chin S.-F., Farooq M., Mejia R., Cronin P. A., Anderson K. S., Kosiorek H. E., Northfelt D. W., Mc Cullough A. E., Patel B. K., Weitzel J. N., Slavin T. P., Caldas C., Pockaj B. A., Murtaza M., Personalized circulating tumor DNA analysis to detect residual disease after neoadjuvant therapy in breast cancer. Sci. Transl. Med. 11, eaax7392 (2019). 31391323 10.1126/scitranslmed.aax7392 PMC7236617

[33] Tie J., Wang Y., Tomasetti C., Li L., Springer S., Kinde I., Silliman N., Tacey M., Wong H.-L., Christie M., Kosmider S., Skinner I., Wong R., Steel M., Tran B., Desai J., Jones I., Haydon A., Hayes T., Price T. J., Strausberg R. L., Diaz L. A. Jr., Papadopoulos N., Kinzler K. W., Vogelstein B., Gibbs P., Circulating tumor DNA analysis detects minimal residual disease and predicts recurrence in patients with stage II colon cancer. Sci. Transl. Med. 8, 346ra92 (2016). 10.1126/scitranslmed.aaf6219 PMC5346159 27384348

[34] Reinert T., Henriksen T. V., Christensen E., Sharma S., Salari R., Sethi H., Knudsen M., Nordentoft I., Wu H.-T., Tin A. S., Heilskov Rasmussen M., Vang S., Shchegrova S., Frydendahl Boll Johansen A., Srinivasan R., Assaf Z., Balcioglu M., Olson A., Dashner S., Hafez D., Navarro S., Goel S., Rabinowitz M., Billings P., Sigurjonsson S., Dyrskjøt L., Swenerton R., Aleshin A., Laurberg S., Husted Madsen A., Kannerup A. S., Stribolt K., Palmelund Krag S., Iversen L. H., Gotschalck Sunesen K., Lin C. H. J., Zimmermann B. G., Lindbjerg Andersen C., Analysis of plasma cell-free DNA by ultradeep sequencing in patients with stages I to III colorectal cancer. JAMA Oncol. 5, 1124–1131 (2019). 10.1001/jamaoncol.2019.0528 PMC6512280 31070691

[35] Khan K. H., Cunningham D., Werner B., Vlachogiannis G., Spiteri I., Heide T., Mateos J. F., Vatsiou A., Lampis A., Damavandi M. D., Lote H., Huntingford I. S., Hedayat S., Chau I., Tunariu N., Mentrasti G., Trevisani F., Rao S., Anandappa G., Watkins D., Starling N., Thomas J., Peckitt C., Khan N., Rugge M., Begum R., Hezelova B., Bryant A., Jones T., Proszek P., Fassan M., Hahne J. C., Hubank M., Braconi C., Sottoriva A., Valeri N., Longitudinal liquid biopsy and mathematical modeling of clonal evolution forecast time to treatment failure in the PROSPECT-C phase II colorectal cancer clinical trial. Cancer Discov. 8, 1270–1285 (2018). 30166348 10.1158/2159-8290.CD-17-0891 PMC6380469

[36] Cescon D. W., Bratman S. V., Chan S. M., Siu L. L., Circulating tumor DNA and liquid biopsy in oncology. Nat. Cancer. 1, 276–290 (2020). 10.1038/s43018-020-0043-5 35122035

[37] Chaudhuri A. A., Chabon J. J., Lovejoy A. F., Newman A. M., Stehr H., Azad T. D., Khodadoust M. S., Esfahani M. S., Liu C. L., Zhou L., Scherer F., Kurtz D. M., Say C., Carter J. N., Merriott D. J., Dudley J. C., Binkley M. S., Modlin L., Padda S. K., Gensheimer M. F., West R. B., Shrager J. B., Neal J. W., Wakelee H. A., Loo B. W. Jr., Alizadeh A. A., Diehn M., Early detection of molecular residual disease in localized lung cancer by circulating tumor DNA profiling. Cancer Discov. 7, 1394–1403 (2017). 28899864 10.1158/2159-8290.CD-17-0716 PMC5895851

[38] NCI Seer, Surveillance, Epidemiology, and End Results (SEER) Research Data 1973–2015 - ASCII Text Data (2018); https://seer.cancer.gov.

[39] Makohon-Moore A. P., Matsukuma K., Zhang M., Reiter J. G., Gerold J. M., Jiao Y., Sikkema L., Yachida M. A. S., Sandone C., Hruban R. H., Klimstra D. S., Papadopoulos N., Nowak M. A., Kinzler K. W., Vogelstein B., Iacobuzio-Donahue C. A., Precancerous neoplastic cells can move through the pancreatic ductal system. Nature 561, 201–205 (2018). 30177826 10.1038/s41586-018-0481-8 PMC6342205

[40] Martincorena I., Campbell P. J., Somatic mutation in cancer and normal cells. Science 349, 1483–1489 (2015). 26404825 10.1126/science.aab4082

[41] McWilliams A., Tammemagi M. C., Mayo J. R., Roberts H., Liu G., Soghrati K., Yasufuku K., Martel S., Laberge F., Gingras M., Atkar-Khattra S., Berg C. D., Evans K., Finley R., Yee J., English J., Nasute P., Goffin J., Puksa S., Stewart L., Tsai S., Johnston M. R., Manos D., Nicholas G., Goss G. D., Seely J. M., Amjadi K., Tremblay A., Burrowes P., MacEachern P., Bhatia R., Tsao M. S., Lam S., Probability of cancer in pulmonary nodules detected on first screening CT. N. Engl. J. Med. 369, 910–919 (2013). 24004118 10.1056/NEJMoa1214726 PMC3951177

[42] The National Lung Screening Trial Research Team, Results of initial low-dose computed tomographic screening for lung cancer. N. Engl. J. Med. 368, 1980–1991 (2013). 23697514 10.1056/NEJMoa1209120 PMC3762603

[43] Clarke C. A., Hubbell E., Kurian A. W., Colditz G. A., Hartman A.-R., Gomez S. L., Projected reductions in absolute cancer–related deaths from diagnosing cancers before metastasis, 2006–2015. Cancer Epidemiol. Biomarkers. Prev. 29, 895–902 (2020). 32229577 10.1158/1055-9965.EPI-19-1366

[44] Douville C., Cohen J. D., Ptak J., Popoli M., Schaefer J., Silliman N., Dobbyn L., Schoen R. E., Tie J., Gibbs P., Goggins M., Wolfgang C. L., Wang T.-L., Shih I. M., Karchin R., Lennon A. M., Hruban R. H., Tomasetti C., Bettegowda C., Kinzler K. W., Papadopoulos N., Vogelstein B., Assessing aneuploidy with repetitive element sequencing. Proc. Natl. Acad. Sci. U.S.A. 117, 4858–4863 (2020). 32075918 10.1073/pnas.1910041117 PMC7060727

[45] The National Lung Screening Trial Research Team, Reduced lung-cancer mortality with low-dose computed tomographic screening. N. Engl. J. Med. 365, 395–409 (2011). 21714641 10.1056/NEJMoa1102873 PMC4356534

[46] de Koning H. J., van der Aalst C. M., de Jong P. A., Scholten E. T., Nackaerts K., Heuvelmans M. A., Lammers J.-W. J., Weenink C., Yousaf-Khan U., Horeweg N., van ’t Westeinde S., Prokop M., Mali W. P., Mohamed Hoesein F. A. A., van Ooijen P. M. A., Aerts J. G. J. V., den Bakker M. A., Thunnissen E., Verschakelen J., Vliegenthart R., Walter J. E., ten Haaf K., Groen H. J. M., Oudkerk M., Reduced lung-cancer mortality with volume CT screening in a randomized trial. N. Engl. J. Med. 382, 503–513 (2020). 31995683 10.1056/NEJMoa1911793

[47] Makohon-Moore A. P., Iacobuzio-Donahue C. A., Pancreatic cancer biology and genetics from an evolutionary perspective. Nat. Rev. Cancer 16, 553–565 (2016). 27444064 10.1038/nrc.2016.66 PMC5739515

[48] Lang B. M., Kuipers J., Misselwitz B., Beerenwinkel N., Predicting colorectal cancer risk from adenoma detection via a two-type branching process model. PLOS Comput. Biol. 16, e1007552 (2020). 32023238 10.1371/journal.pcbi.1007552 PMC7001909

[49] de Koning H. J., Meza R., Plevritis S. K., ten Haaf K., Munshi V. N., Jeon J., Erdogan S. A., Kong C. Y., Han S. S., van Rosmalen J., Choi S. E., Pinsky P. F., de Gonzalez A. B., Berg C. D., Black W. C., Tammemägi M. C., Hazelton W. D., Feuer E. J., McMahon P. M., Benefits and harms of computed tomography lung cancer screening strategies: A comparative modeling study for the U.S. Preventive Services Task Force. Ann. Intern. Med. 160, 311–320 (2014). 24379002 10.7326/M13-2316 PMC4116741

[50] Curtius K., Dewanji A., Hazelton W. D., Rubenstein J. H., Luebeck E. G., Optimal timing for cancer screening and adaptive surveillance using mathematical modeling. bioRxiv, 2020.02.11.927475 (2020). 10.1158/0008-5472.CAN-20-0335 PMC7611607 33293425

[51] Hori S. S., Lutz A. M., Paulmurugan R., Gambhir S. S., A model-based personalized cancer screening strategy for detecting early-stage tumors using blood-borne biomarkers. Cancer Res. 77, 2570–2584 (2017). 28283654 10.1158/0008-5472.CAN-16-2904 PMC6010058

[52] K. B. Athreya, P. E. Ney, *Branching Processes* (Springer-Verlag, Berlin Heidelberg, 1972).

[53] L. Allen, *An Introduction to Stochastic Processes with Applications to Biology* (Boca Raton, FL: Chapman & Hall/CRC, ed. 2, 2011).

[54] V. G. Kulkarni, *Modeling and Analysis of Stochastic Systems* (Chapman and Hall/CRC, 2016).

[55] Cheek D., Antal T., Mutation frequencies in a birth-death branching process. Ann. Appl. Probab. 28, 3922–3947 (2018).

[56] D. J. Daley, D. Vere-Jones, *Introduction to the Theory of Point Processes* (Springer New York, 2006).

[57] Antal T., Krapivsky P. L., Exact solution of a two-type branching process: Clone size distribution in cell division kinetics. J. Stat. Mech. Theory Exp. 2010, P07028 (2010).

[58] Antal T., Krapivsky P. L., Exact solution of a two-type branching process: Models of tumor progression. J. Stat. Mech. Theory Exp. 08, P08018 (2011).

[59] A. Ronveaux, *Heun’s Differential Equations* (Oxford Univ. Press, 1995).

[60] O. V Motygin, in *2018 Days on Diffraction (DD)* (IEEE, 2018), pp. 223–229.

[61] M. Abramowitz, I. A. Stegun, *Handbook of Mathematical Functions: With Formulas, Graphs, and Mathematical Tables* (Applied mathematics series, Dover Publications, 1964).

[62] Choudhury G. L., Lucantoni D. M., Whitt W., Multidimensional transform inversion with applications to the transient M/G/1 queue. Ann. Appl. Probab. 4, 719–740 (1994).

