# Supplementary material for: A mathematical model of ctDNA shedding predicts tumor detection size
Source: Sci Adv. 2020 Dec 11;6(50):eabc4308. doi: 10.1126/sciadv.abc4308 (PMC7732186; doi:10.1126/sciadv.abc4308)
Supplement: http://advances.sciencemag.org/cgi/content/full/6/50/eabc4308/DC1 [file supp_6_50_eabc4308__index.html]

Science Advances | Science AdvancesAAASSearchScience AdvancesMenu

## Supplementary Materials

# A mathematical model of ctDNA shedding predicts tumor detection size

Stefano Avanzini, David M. Kurtz, Jacob J. Chabon, Everett J. Moding, Sharon Seiko Hori, Sanjiv Sam Gambhir, Ash A. Alizadeh, Maximilian Diehn, Johannes G. Reiter

Download Supplement

**The PDF file includes:**

- Materials and Methods
- Figs. S1 to S13
- Tables S1 to S3
- Note S1
- References

**Other Supplementary Material for this manuscript includes the following:**

- Movie S1

**Files in this Data Supplement:**

- Adobe PDF - abc4308\_SM.pdf
- abc4308\_Movie\_S1.mp4
